# Optimized Liquid-Phase Exfoliation of Magnetic van der Waals Heterostructures: Towards the Single Layer and Deterministic Fabrication of Devices

**DOI:** 10.3390/molecules26237371

**Published:** 2021-12-04

**Authors:** Lucía Martín-Pérez, Enrique Burzurí

**Affiliations:** 1 IMDEA Nanociencia, Campus de Cantoblanco, Calle Faraday 9, 28049 Madrid, Spain; lucia.martin@imdea.org; 2 Departamento de Física de la Materia Condensada, Universidad Autónoma de Madrid, 28049 Madrid, Spain

**Keywords:** cylindrite, van der Waals, liquid phase exfoliation

## Abstract

Van der Waals magnetic materials are promising candidates for spintronics and testbeds for exotic magnetic phenomena in low dimensions. The two-dimensional (2D) limit in these materials is typically reached by mechanically breaking the van der Waals interactions between layers. Alternative approaches to producing large amounts of flakes rely on wet methods such as liquid-phase exfoliation (LPE). Here, we report an optimized route for obtaining monolayers of magnetic cylindrite by LPE. We show that the selection of exfoliation times is the determining factor in producing a statistically significant amount of monolayers while keeping relatively big flake areas (~1 µm^2^). We show that the cylindrite lattice is preserved in the flakes after LPE. To study the electron transport properties, we have fabricated field-effect transistors based on LPE cylindrite. Flakes are deterministically positioned between nanoscale electrodes by dielectrophoresis. We show that dielectrophoresis can selectively move the larger flakes into the devices. Cylindrite nanoscale flakes present a p-doped semiconducting behaviour, in agreement with the mechanically exfoliated counterparts. Alternating current (AC) admittance spectroscopy sheds light on the role played by potential barriers between different flakes in terms of electron transport properties. The present large-scale exfoliation and device fabrication strategy can be extrapolated to other families of magnetic materials.

## 1. Introduction

Layered magnetic materials [1,2,3] are emerging as the latest acquisition of the van der Waals (vdW) materials family and their heterostructures [4,5]. An increasingly growing number of reports demonstrate exfoliation of the bulk and persistence of the magnetism down to the monolayer limit. Some pioneering examples, where magnetism at the 2D limit is unequivocally proven, are ferromagnetic CrI_3_ [6,7], CrGeTe_3_ [8], Fe_3_GeTe_2_ [9], VSe_2_ [10], and the antiferromagnetic FePS_3_ [11], among other examples. Magnetism is intrinsically linked to the dimensionality of the materials, and, therefore, cleavable magnetic materials are particularly interesting testbeds to study a plethora of magnetic phenomena that may appear or be quenched at the 2D limit; for example, magnetic order, strong spin fluctuations, skyrmions, and new quantum phases [1,3]. From a more applied perspective, the combination of conductivity and magnetism in a robust material may be the determining factor in pushing forward a new generation of spintronics devices [9,12].

The most widespread method to obtain thin flakes of vdW materials, from few to a single layer, is the mechanical exfoliation of the bulk compound; that is, the repeated peeling of bulk material with an adhesive tape that breaks the van der Waals forces between layers [13]. This method results in clean flakes, their placement in electrical devices, and the formation of heterostructures by deterministic mechanical stamping [14,15]. The main limitation of this method is the low throughput in obtaining flakes and the fabrication of devices.

Alternative approaches able to produce large amounts of flakes rely on wet methods that use different agents to separate layers: ionic-intercalation [16,17,18,19,20], ionic-exchange [21,22,23,24], electrochemical [25,26,27,28], and liquid-phase exfoliation [29,30,31]. The flakes obtained via these methods are typically dispersed in a liquid media and display a distribution of thickness and area. Centrifugation of the resultant material can be used to remove the thicker material and further narrow down the statistical distribution to a specific thickness [30,32,33,34] linked to the volume of the flakes.

**Figure 1:**
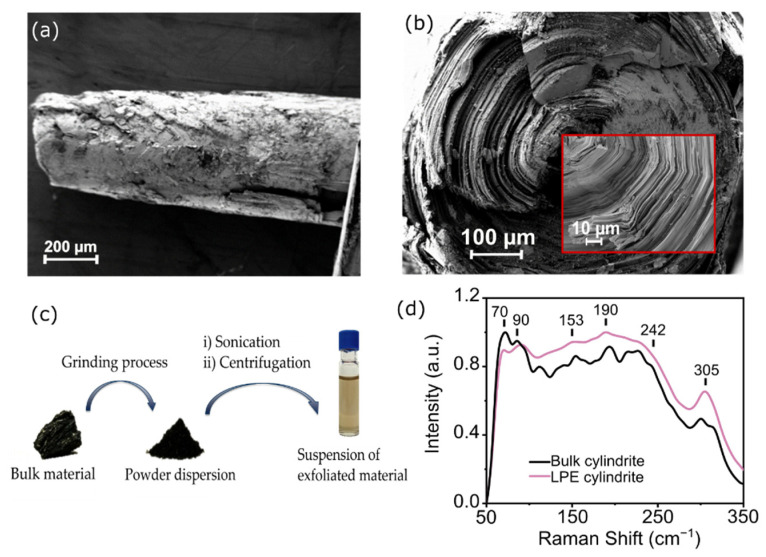
(**a**) Scanning Electron Microscopy (SEM) image of an individual cylindrite cylinder and (**b**) its transverse-plane. The inset shows an amplified region around the revolution axis of the cylinder. (**c**) Schematic of the two-step Liquid Phase Exfoliation (LPE) process. Starting from the bulk mineral, some cylinders are extracted and ground in an agate mortar until a black powder is obtained. The powder is dispersed in *i*PrOH at a 1 mg·mL^−1^ concentration and is sonicated in an ultrasonic bath and centrifuged in order to eliminate not-exfoliated flakes. (**d**) Normalized Raman spectra (λ_exc_ = 532 nm) of cylindrite bulk (black line) and LPE cylindrite obtained after sonication of a 1 mg·mL^−1^ powder dispersion in *i*PrOH (purple line).

Very recently, the mechanical exfoliation of a naturally occurring van der Waals heterostructure was reported: cylindrite [35]. Cylindrite is a member of the sulfosalt family of minerals with the formula Pb_3_Sn_4_FeSb_2_S_14_ [36,37]. The heterostructure is formed by alternating SnS_2_ and PbS layers with octahedral (O) and pseudo-tetragonal (T) symmetry, respectively. The sheets are held together by van der Waals interactions and rolled into triclinic pinacoidal crystals (cylinders). Figure 1a,b shows two scanning electron microscope (SEM) images of a single cylindrite crystal. The layered structure and the cylindrical shape are clearly observed in the images. See Supplementary Figures S1–S4 for additional SEM images. Bulk cylindrite presents ferromagnetic interactions and a magnetic ordered phase due to the substitutional Fe atoms present in the lattice [35,36,37]. Interestingly, a preliminary attempt of liquid-phase exfoliation showed that the magnetic interactions are preserved in thinner or smaller flakes with slightly decreased transition temperature [35].

However, the average flake thickness and topology resultant from LPE was not reported nor statistically analyzed. This knowledge is crucial when studying magnetism at the boundary between two and three dimensions. LPE of a similar vdW heterostructure, franckeite, yielded a thickness of several tens of nanometers [38], considerably lower than bulk but still far from the monolayer limit. Moreover, the structural stability and electrical properties of the cylindrite flakes after the extreme LPE conditions was not explored.

Here we report an optimized route for obtaining cylindrite monolayers by LPE. We show that the selection of exfoliation times and the starting material are determinant to consistently obtain monolayers. A careful topological analysis shows that a three hour sonication of the bulk material produces a statistically significant amount of monolayers while keeping relatively large flake areas (~1 µm^2^), in contrast with previous reports [35]. We show that the cylindrite lattice is preserved in the flakes after LPE. To study the electron transport properties, we have fabricated field-effect transistors based on LPE cylindrite. Flakes are deterministically positioned between nanoscale electrodes by dielectrophoresis (DEP). We show that DEP can selectively move the larger flakes into the devices. Cylindrite nanoscale flakes present a p-doped semiconducting behavior, in agreement with the mechanically exfoliated counterparts. AC admittance spectroscopy gives light to the role played by potential barriers between different flakes in electron transport properties.

## 2. Results

### 2.1. Liquid Phase Exfoliation of the Heterostructures. Structural Characterization

Figure 1c shows the different LPE steps carried out for obtaining cylindrite flakes. First, several cylindrite cylinders are extracted from the bulk material and ground in an agate mortar until a black powder is obtained. Thereafter, the cylindrite powder is mixed with *i*PrOH as a solvent. LPE in *i*PrOH proceeds smoothly without the help of any addition of surfactant species or any washing step, as previously reported [35,38]. In addition, its relatively weak polarizability and high boiling point facilitates the fabrication of devices by DEP [38]. The suspension of LPE cylindrite nanoflakes in *i*PrOH is obtained via a two-step process: (i) 1 h sonication of 1 mg·mL^−1^ dispersion of cylindrite powder in *i*PrOH in an ultrasonic bath kept at 20 °C; followed by (ii) 30 min centrifugation of the as-prepared suspension (990 g, 20 °C, Beckman Coulter Allegra X-15R, FX6100 rotor, radius 9.8 cm) in order to discard thicker or non-exfoliated flakes. After this process, the supernatant is collected to obtain a pale orange-colored exfoliated cylindrite suspension.

The resultant suspension of LPE cylindrite nanoflakes is characterized by Raman spectroscopy to ensure the integrity of the heterostructure after the overall exfoliation process. Figure 1d shows the Raman spectra of both bulk and a representative exfoliated nanoflake drop-casted onto a silicon wafer. Both samples present similar spectra, with six main bands centered at 70, 90, 153, 190, 228, and 305 cm^−1^. These Raman bands can be associated to the SnS_2_ and PbS substructures that form cylindrite (See Supplementary Table S1). The band at 70 cm^−1^ can be assigned to acoustic phonon modes of the PbS layer [38,39,40]. The bands at 305 cm^−1^ and 90 cm^−1^ are associated to the A_1g_ mode of SnS_2_ and SnS layers respectively [38,39,40,41]. The band at 153 cm^−1^ results from a combination of the 2nd order effect of the SnS_2_ layers and the transverse optical and acoustic modes phonon modes of the PbS layers. The band at 190 cm^−1^ is also a combination of the longitudinal optical phonon mode of the PbS layer and the atomic orbital E_g_ of the SnS_2_ layers [38,39,40]. These results confirm the integrity of the heterostructure after the LPE sonication plus centrifugation cycle.

In addition, the Raman spectrum of the bulk material presents a shoulder at 242 cm^−1^ assigned to a combination of the phonon modes of both layers, its intensity being an indication of the thickness of the heterostructure [39]. This band appears diminished in the spectrum of the exfoliated cylindrite, which may be an indication of a significant decrease in the thickness of the flake with respect to the starting material.

### 2.2. Topological Statistical Characterization. Optimization of the LPE Process

The morphology and size distribution of the LPE flakes is studied by atomic force microscopy (AFM) and scanning electron microscopy (SEM). Samples are prepared by spin-coating of the corresponding dispersion on a silicon wafer or mica foil and dried in air (see Methods).

**Figure 2:**
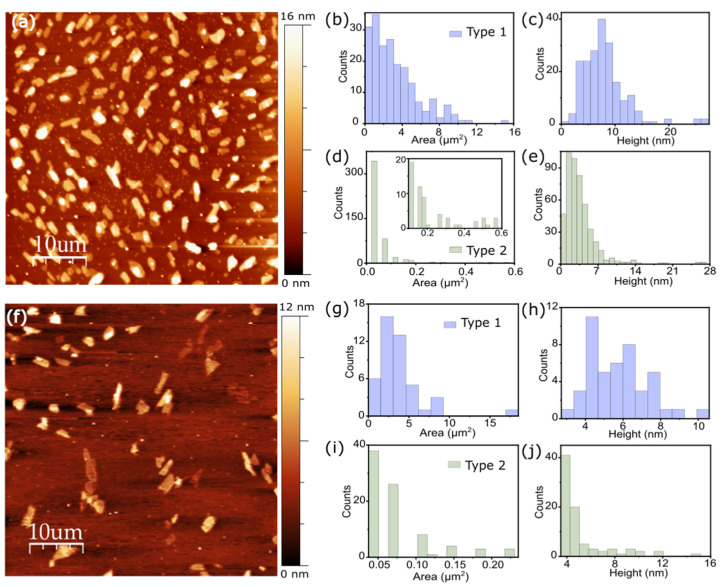
Atomic force microscopy (AFM) study of LPE cylindrite flakes. (**a**) AFM topographic characterization of cylindrite nanoflakes obtained in Sample 1. Image dimension: 50 × 50 µm^2^. Statistical analysis of AFM area and height data of type 1 (**b**,**c**) and type 2 (**d**,**e**) cylindrite nanoflakes. (**f**) AFM topographic characterization of cylindrite nanoflakes obtained in Sample 3. Image dimension: 50 × 50 µm^2^. Statistical analysis of AFM area and height data of type 1 (**g**,**h**) and type 2 (**i**,**j**) cylindrite nanoflakes.

Figure 2a shows the AFM topographic characterization of the cylindrite nanosheets obtained in Sample 1. Two distinct families of flakes can be observed in the image: a group of flakes with larger areas (type 1 family) and an additional numerous group of flakes with substantially smaller areas (type 2 family) (see Supplementary Materials for details about the flake division in groups). Figure 2b–e shows the statistical analysis performed on each population on a 50 × 50 µm^2^ area AFM image (Figure 2a). The type 1 family presents flake areas of more than 1 μm^2^. The average thickness of the flakes is 7.2 nm; far, therefore, from the monolayer limit. On the other hand, type 2 flakes present average thicknesses of around 2.4 nm; closer, therefore, to the monolayer limit. However, the average flake area is much smaller, mostly centered at 0.05 µm^2^ and not exceeding 0.6 µm^2^. Type 2 flakes, with thicknesses close to the monolayer, correspond to less than 5% of the total exfoliated area (see Supplementary Figures S5–S12 for details).

An additional re-exfoliation attempt is carried out to try to thin down the type 1 flake thickness. For this, Sample 1 exfoliated material is subject to a second LPE under the same conditions (Sample 2). Strikingly, the topological study carried out on Sample 2 shows no clear morphology of the flakes (see Figures S13–S18 in the Supplementary Materials). This may occur due to the partial degradation of the layers by oxidation processes happening between the two exfoliation steps, or by reaggregation processes, since the initial exfoliated samples were not stabilized with any external agent so the flakes together with the solvent molecules could recombine [42,43]. This may prevent further exfoliation or an accurate topological analysis in AFM.

An alternative path that may provide thinner flakes is to increase the initial sonication and centrifugation times of freshly molted cylindrite. Two new batches of LPE cylindrite flakes are prepared by following the same method but increasing the sonication time to 2 and 3 h (Samples 3 and 5 respectively) and the centrifugation time to 1 h. Table 1 summarizes the different Samples and corresponding sonication and centrifugation times.

**Figure 3:**
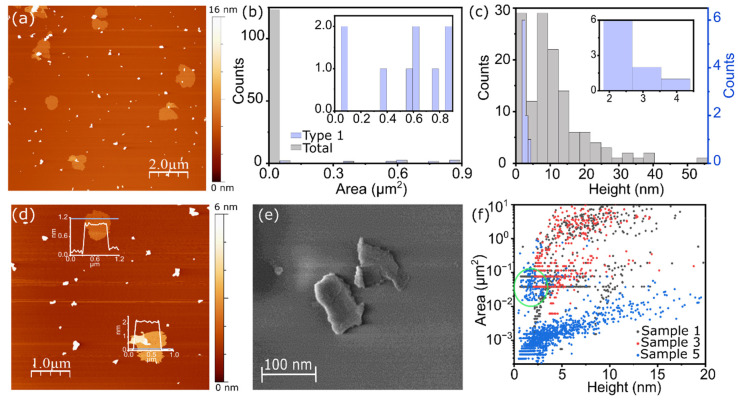
AFM study of LPE cylindrite flakes. (**a**) AFM topographic characterization of cylindrite nanoflakes obtained in Sample 5. Image dimension: 10 × 10 µm^2^. Statistical analysis of AFM (**b**) area and (**c**) height of cylindrite nanoflakes. The inset graphics show the distribution of type 1 flakes in each case. (**d**) AFM topographic characterization of cylindrite nanoflakes with the corresponding height profile of two type 1 flakes. Image dimension: 5 × 5 µm^2^. (**e**) SEM image of the cylindrite nanosheets obtained in Sample 5. (**f**) Scatter plot of area and height data of Sample 1 (gray), Sample 3 (red), and Sample 5 (blue) LPE cylindrite nanosheets obtained through an AFM study.

Figure 3a shows the AFM topographic characterization of the cylindrite nanosheets obtained in Sample 5. Two distinct families of flakes with larger (type 1) and smaller (type 2) areas are observed, as in Sample 1 in Figure 2 (see Supplementary Figures S19–S22 for details). Figure 3b,c shows the statistical analysis of the thickness and flake area. Larger type 1 flakes present areas mostly between 0.5–0.9 µm^2^. Larger sonication and centrifugation times tend therefore to induce a larger breaking and filtering of the bigger flakes. This significantly decreases the average area of the flakes when compared to Sample 1. This area difference notwithstanding, the lateral size of the flakes is still large enough for typical nanoscale devices, as seen below [38]. Interestingly, the thickness of all type 1 flakes lies below 4 nm, which is consistently close to the monolayer limit. The larger sonication and centrifugation times therefore lead to a more consistent exfoliation of the material in the nanometer scale, discarding the larger and thicker flakes. On the other hand, smaller type 2 flakes, with areas mostly around 0.02–0.03 μm^2^ present an average thickness of 8 nm with a broader distribution between 1 nm (around the monolayer limit) and up to 40 nm.

Figure 3d shows an AFM image of two representative type 1 nanosheets (sample 5) with their corresponding height profiles. The average height of the flakes ranges between 1 and 2 nm. A representative SEM image of the flakes is shown in Figure 3e. The single unit cell in cylindrite is made of a SnS_2_-PbS (O-T) pair with a total height of ~1.18 nm. In addition, moisture trapped between the layers and the substrate, or adsorbed solvent molecules, tend to introduce an offset in the AFM topography of about 1.2–1.3 nm [31,40]. Accordingly, the expected height of the monolayers in AFM would be in the 1–2.5 nm range. The flakes obtained in Sample 5 are therefore cylindrite monolayers.

The fine tuning of the sonication time provides a handle with which to gradually shift from the 2D to the 3D limit. A comparative topological and statistical analysis performed in Sample 3 flakes (Figure 2f–j) with intermediate sonication time shows that the average area (2.9 µm^2^) and thickness (5.3 µm) of the type 1 flakes lies between those in Sample 1 and Sample 5 (see Supplementary Materials Figure S23 for a clear direct comparison). Figure 3f shows the area–thickness scatter plot of the flakes obtained from different AFM images in Samples 1, 3, and 5. The different populations of flakes obtained in each Sample can be clearly differentiated. Sample 5 produces a sizable population of large flakes with thicknesses below the 4 nm (green circle), whereas Sample 1 produces larger flakes but with larger average thickness.

### 2.3. Electron Transport Characterization

**Figure 4:**
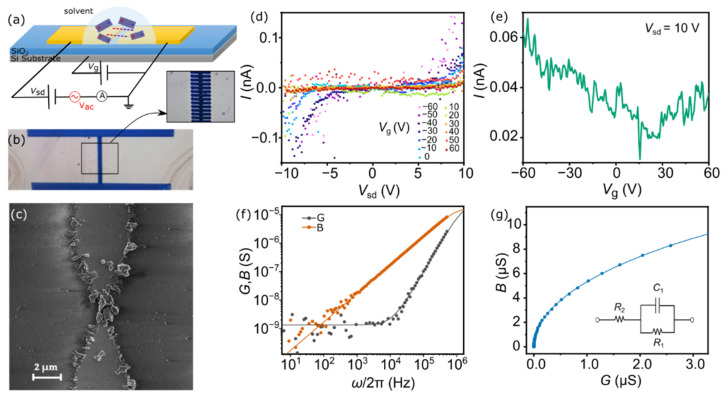
DEP assembly of LPE cylindrite. (**a**) Schematic representation of the DEP of colloidal heterostructures between metallic electrodes. (**b**) Optical image of a multi-electrode device. (**c**) SEM image of a single pair of electrodes after DEP. Several cylindrite flakes bridge the gap between the electrodes. (**d**) Current–voltage (*I–V*_sd_) characteristic measured at different gate voltages after DEP assembly. (**e**) Current as a function of the applied back-gate voltage *V*_g_ at a fixed *V*_sd_ = 10 V. (**f**) *G* and *B* frequency spectrum measured on a cylindrite-based FET. Solid lines are *G* and *B* simulations using a *R*_2_-*C*_1_*R*_1_ model (see text). (**g**) Nyquist representation of the complex admittance. The inset shows the equivalent *R-CR* circuit used to model the admittance.

The electrical properties of the exfoliated cylindrite flakes were explored in a solid-state field-effect transistor (FET) geometry. Tens of finger-like electrodes connected to a common contact pad are fabricated by mask-less laser lithography and subsequent evaporation of Cr/Au on a Si/SiO_2_ substrate (see Methods for additional fabrication details). The Si substrate is used as a back gate electrode and the SiO_2_ as the gate dielectric that separates gate from source/drain electrodes (see Figure 4a,b for schematics and an optical image of a representative device and a zoom-in area around the electrode tips). The gap between source and drain electrodes is set to 1–1.5 µm.

The exfoliated cylindrite flakes are positioned between the electrodes by dielectrophoresis (DEP) [44,45,46,47]. Dielectrophoresis has been recently used to position nanostructures such as nanoparticles [46,47], carbon nanotubes [48,49,50], and 2D materials [38] in specific areas of nanoscale devices with high precision. In short: a droplet containing the exfoliated and dispersed cylindrite flakes (Sample 5) is placed on top of a device, covering the inter-electrode area; subsequently, an AC voltage is applied between the tips of the electrodes thus generating an AC electrical field. The specifically designed tip-ended electrodes (see Figure 4b) focus the field and maximize the electrical gradient towards the central area. The dielectrophoretic force acting on a prolate ellipsoid is:

(1) $$F_{DEP}\propto V_{p}\varepsilon_{m}Re\left[\frac{\varepsilon_{p}^{*}-\varepsilon_{m}^{*}}{\varepsilon_{m}^{*}}\right]\nabla {\left|E\right|}^{2}$$

where *Vp* is the volume of the particle, *E* is the electrical field, $Re\left[\frac{\varepsilon_{p}^{*}-\varepsilon_{m}^{*}}{\varepsilon_{m}^{*}}\right]$ is the Clausius-Mossotti factor and ε_p_* and ε_m_* are the complex permittivity of the particle and the media respectively. The sign of the dielectrophoretic force depends on the relative dielectric properties of the material and the solvent. At high frequencies, typically used in DEP, the CM factor can be approximated to the real components of the permittivity such that $Re\left[\frac{\varepsilon_{p}-\varepsilon_{m}}{\varepsilon_{m}}\right]$. In addition, the CM factor can be expressed for convenience as a function of the polarizability of particle and medium: $\alpha \sim \frac{\varepsilon -1}{\varepsilon +2}$. A proper combination of these parameters polarizes the dispersed particles (cylindrite) in the liquid media and transports them along the gradient direction. The DEP force will transport and align cylindrite toward the gap between the electrodes only if the flakes are more polarizable than the surrounding medium. Isopropanol, with relatively weak polarizability (α = 6.98 Å^3^), has proven to be a good candidate to transport similar van der Waals heterostructures with the same constituent sub-layers (α ≈ 10 Å^3^) [38]. Different sets of parameters have been explored to maximize the probability of having a continuous path of cylindrite flakes between the electrodes without saturating the electrode area with flakes (see Supplementary Materials for additional images). The optimized parameters are **V*_AC_* = 10 V, ν = 1 MHz and t = 10 min. These values are higher than those typically reported for carbon nanotubes, as expected for the different aspect ratios between flakes and carbon nanotubes [48,49]. The sample is annealed in a vacuum to remove remnants of the solvent.

Figure 4c shows a scanning electron microscope (SEM) image of a representative device after dielectrophoresis. Several cylindrite flakes bridge the gap between the electrodes forming a continuous path between them (see Supplementary Materials Figures S24–S25 for additional SEM images). In addition to this, additional flakes can be seen attached along the edge of the electrodes as a result of electrical field gradients with the substrate [38]. The later disconnected flakes do not participate in charge transport since charge carriers cannot go from one electrode to the other across those flakes.

Interestingly, the two families of flakes obtained by LPE and identified on the AFM images (Figure 2 and Figure 3) behave radically differently under the DEP process. On the one hand, the majority of flakes in the gap and decorating the electrodes edges are type 1 flakes. That is, flakes with thicknesses below 4 nm and approximately 0.1–0.9 µm^2^ in area (see Figure 4c). In addition, the silicon substrate is free of these type 1 flakes (see Supplementary Materials for large-area SEM images). On the other hand, the smaller type 2 flakes are homogenously scattered over the SiO_2_/Si substrate. In other words, type 2 flakes seem not to be subject to the dielectrophoretic force.

The rationale behind this selectivity can be understood by exploring the nature of the dielectrophoretic force in Equation (1). Larger aspect-ratio flakes (type 1) will be more similar to prolate ellipsoids where Equation (1) holds valid, whereas smaller aspect-ratio flakes (type 2) are closer to spherical flakes. The electrical field induces stronger dipoles, therefore, in type 1 flakes than in type 2. In addition, the volume of type 2 flakes is significantly smaller than that of type 1 flakes. This translates into a lower dielectrophoretic force that may not be able to overcome Brownian motion of the small flakes in the liquid media, as recently described [47]. DEP positioning is therefore able to selectively move thinner and larger area flakes into specific areas of nanoscale devices.

Figure 4d shows the current (*I*) measured as a function of the source-drain voltage (*V*_sd_) and at different gate voltages (*V*_g_) applied to the Si substrate. The current shows the s-shape behavior characteristic of semiconducting materials with non-ohmic contacts when in contact with the Au electrodes. This result proves the formation of a continuous charge transport path across the cylindrite bridge when compared with the empty device. Current levels in different devices are found in the order of 0.25–50 nA, that is, lower than in mechanically-exfoliated cylindrite [35]. This is consistent with previous reports on LPE van der Waals heterostructures such as franckeite [38]. Of note is that the LPE cylindrite flakes are, on average, significantly thinner than those reported in previous studies [35].

Figure 4e shows the gate trace measured at a fixed *V*_sd_ = 10 V. The current decreases by increasing *V*_g_ to more positive values. This behavior is characteristic of p-doped materials and is qualitatively in agreement with the electrical characterization performed on mechanically-exfoliated cylindrite flakes [35]. This result shows that the LPE process and the presence of solvent adsorbates does not significantly alter the main electronic properties of the flakes.

Frequency dependent AC admittance spectroscopy has been performed to understand the role played by grain boundaries and contacts between flakes in the charge transport across multi-flake cylindrite FETs. The complex admittance Y* = *G* + *iB*, where *G* and *B* are the conductance and susceptance respectively, is measured by using a lock-in technique. Figure 4f shows *G* and *B* measured as a function of frequency (5 Hz < ω/2π < 510 kHz) and a fixed AC excitation (*V*_AC_ = 100 mV) and DC offset (*V*_off_ = 5 V) (see Methods for additional details). A monotonic increment in *B* is observed as a function of the frequency whereas *G* remains roughly constant at low frequencies and increases monotonically at high frequencies. This trend points to the presence of resistive *R* and capacitive *C* elements in the circuit. The admittance (or impedance) of multigrain materials is typically modelled by two parallel *RC* circuits that account for two contributions: the grain boundaries and the bulk [51]. In this particular case, the results can be quantitatively reproduced by a single parallel *RC* circuit connected in series to a single *R* (see inset in Figure 4g); that is, one of the capacitances is negligible in the frequency range of the measurement. The solid lines in Figure 4f are *G* and *B* simulated for an *R_-_CR* equivalent circuit with values *R*_1_ = 750 MΩ, *C*_1_ = 17 pF, *R*_2_ = 34 kΩ (see Supplementary Materials for a detailed description of the model).

Figure 4g shows the Nyquist representation of the admittance and the correspondent simulation with the same *R*_1_, *C*_1_, and *R*_2_ parameters (see Supplementary Materials). The absence of complete semicircles in the frequency range prevents the univocal assignment of the *R*_1_, *C*_1_, and *R*_2_ values whether to grain boundaries or to the bulk. It can be assumed that the larger resistance will correspond to internal grain boundaries [51]. Nevertheless, the results show that charge transport in the nanoscale devices is governed by resistive grain boundaries with negligible capacitive contributions.

## 3. Materials and Methods

### 3.1. Chemicals and Reagents

The bulk cylindrite mineral was obtained from the San José, Oruro (Bolivia) mine. The same crystal is used for all experiments. The solvent (*i*PrOH) used for the LPE process is purchased from Scharlab Chemicals S. L (Madrid, Spain). and used without further purification.

### 3.2. Experimental Procedures and Equipment

- Preparation of franckeite colloidal suspension.

Cylinders from natural cylindrite are ground in an agate mortar until a fine black powder is obtained. Cylindrite powder (1 mg) is dispersed in *i*PrOH (1 mL) in a 20 mL glass vial. The dispersion is subjected to ultrasound irradiation for different times in an ultrasonic bath (Fisher Scientific (Hampton, NH, USA) FB 15051; 37 kHz, 280 W, ultrasonic peak max. 320 W, standard sine wave modulation) connected to a cooling system maintaining the water bath temperature at 20 °C. The resulting black suspension is centrifuged at 990 g and 20 °C for different times (Beckman Coulter (Brea, CA, USA) Allegra X-15R, FX6100 rotor, radius 9.8 cm); it separated into a black sediment and a pale-orange supernatant, which is carefully iso-lated from the solid. The corresponding cylindrite suspension remained colloidally stable for 48 h to 72 h, after which it progressively deposited. Nonetheless, the cylindrite flakes could easily be re-dispersed by a 1–2 min bath sonication.

- Raman spectroscopy

The colloidal samples are drop-cast and dried onto a silicon wafer at 50 °C. Their Raman spectra are recorded with a Bruker Senterra confocal Raman microscope (Bruker Optic, Ettlingen, Germany, resolution 3–5 cm^–1^) using the following parameters: objective NA 0.75, x50; laser excitation: 532 nm, 0.2 mW, 5 coadditions. The spectra result from the average of several measurements acquired from different regions over the whole samples.

- Atomic Force Microscopy (AFM)

Dried AFM samples are prepared by spin-coating (Laurell Technologies (North Wales, PA, USA), WS-400BZ-6NPP/LITE Spin Coater) of the corresponding dispersion on a silicon wafer (Sample 1–4) or mica foil (Sample 5) and dried in air. AFM images are acquired using commercial AFM systems (NT-MDT Ntegra Prima and JPK Nanowizard 2) in semicontact (dynamic) mode in ambient conditions. NT-MDT NSG01 silicon cantilevers, with typical values of 5.1 N·m^−1^ spring constant and 150 kHz resonant frequency are employed in all cases. The resulting images are processed using WSxM software [52] (version 5.0, Nanotec Electronica S.L., Tres Cantos, Spain) and the statistical analysis is done using Gwyddion software.

- Dielectrophoretic process (DEP)

DEP technique is performed in a Lakeshore Cryogenics (Westerville, OH, USA) (Model PS-100 Tabletop) probe station, equipped with a FeelTech FY3200S Dual-channel Arbitrary Function Signal Generator, applying the following parameters: **V*_AC_* = 10 V, ν = 1 MHz, and *t* = 10 min.

- Scanning electron microscopy (SEM)

The SEM images are recorded by a secondary electron detector mounted in a Carl Zeiss AURIGA Scanning Electron Microscope (Stuttgart, Germany). The acceleration voltage is 2 kV and the working distance is 8 mm.

- Optical microscopy images of DEP devices

Optical microscopy images of the DEP devices are acquired with a Lakeshore Cryo-genics (Model PS-100 Tabletop) probe station Z70 microscope vision system equipped with an STC-HD93DV (Omron Sentech, (Kanagawa, Japan)) camera.

- Electron Transport Measurements

The current–voltage curves and gate characteristics are obtained in ambient conditions in the chamber of a Lakeshore Cryogenics (Model PS-100 Tabletop) probe station, equipped with a Keithley 2450 digital source-meter unit and a Tenma 72-270 Programmable DC Power Supply (60 V, 3 A)

- Frequency dependent AC admittance spectroscopy

AC admittance measurements as a function of the frequency are performed in a Lakeshore Cryogenics (Model PS-100 Tabletop) probe station, equipped with a Zurich In-struments (Zurich, Switzerland) MLI 500 kHz/5 MHz Lock-in Amplifier. Measurement parameters: frequency (5 Hz < ω/2π < 510 kHz) and a fixed AC excitation (*V*_AC_ = 100 mV) and DC offset (*V*_off_ = 5 V).

- Device fabrication details

The multi-electrode devices are fabricated via laser mask-less optical lithography and thermal evaporation of Cr/Au (5/80 nm) electrodes on a highly-doped silicon substrate capped with a 300 nm thick insulating SiO2 layer, used as common back-gate electrode. Initially, Si/SiO2 wafers are cleaned using *i*PrOH and acetone to remove any traces of organic, ionic, and metallic impurities. Then, AZ1505 positive photoresist is spin coated at 5000 rpm for 1 min onto the surface followed by baking at 90 °C for 1 min to form a 450 nm resist layer. The electrodes and pads are defined by exposing the surface to UV light using a Heidelberg Instruments (Heidelberg, Germany) DWL66fs laser writer of 405 nm (h-line) with 300 mJ/cm^2^ dose. The pattern is subsequently developed with AZ-351B. Thereafter 5 nm Cr and 80 nm Au layers are deposited using Ecovac e-beam evaporation by Angstrom Engineering (Kitchener, Canada). A lift-off process in acetone/*i*PrOH/deionized water removes the excess metallic material. The finger-shaped electrodes are connected to common Au pads that allow simultaneous dielectrophoresis on all the devices. The size of the gap created between a pair of electrodes is 1 µm. The devices are annealed at 300 °C for 8 h after the fabrication.

## 4. Conclusions

In conclusion, we report an optimized route and general guidelines for liquid phase exfoliation of the magnetic cylindrite. The fine tuning of sonication and centrifugation times can be used to obtain a range of flake areas and thicknesses. We show that this approach provides flakes down to the monolayer limit with average areas of 1 µm^2^. This control is fundamental for studying magnetism at the boundary between 2D and 3D. The topological and structural analysis shows that the cylindrite lattice is preserved after LPE. We show that a proper combination of DEP parameters is selective of the thinner and larger area cylindrite flakes and transport them into nanoscale devices. The electrical properties are consistent with a p-doped semiconducting material. This shows that a combined LPE + DEP approach allows the fabrication of cylindrite-based flakes with properties equivalent to mechanically-exfoliated counterparts. AC admittance spectroscopy shows that the electronic behavior of the cylindrite-based FETs is mainly determined by resistivity while capacitive effects are negligible, especially at low-medium frequencies. The present large-scale exfoliation and device fabrication strategy can be extrapolated to other families of magnetic materials.

## Figures and Tables

**Table 1:** Summary of Samples and their corresponding sonication and centrifugation times. Samples 2 and 4 are obtained after the re-exfoliation of previous Samples 1 and 3, respectively.

	Sonication Time	Centrifugation Time
Sample 1	1 h	30 min
Sample 2	(1 h) + 30 min	(30) min + 30 min
Sample 3	2 h	1 h
Sample 4	(2 h) + 1 h	(1 h) + 1 h
Sample 5	3 h	1 h

## Data Availability

The data presented in this study are available on request from the corresponding author.

## Supplementary Materials

Figures S1–S4: Additional Scanning Electron Microscopy (SEM) images of bulk cylindrite. Table S1: Interpretation of the Raman spectra. Figures S5–S22: additional AFM images of different exfoliation conditions. Figure S23: Statistics on type 1 flakes in Sample 5. Figures S24 and S25: Additional SEM images of on-device cylindrite flakes. Section S9: Mathematical description of the R-CR electrical model.

## Sample Availability

Samples of the compounds are not available from the authors.
