# Supplementary material for: Optimized Liquid-Phase Exfoliation of Magnetic van der Waals Heterostructures: Towards the Single Layer and Deterministic Fabrication of Devices
Source: Molecules. 2021 Dec 4;26(23):7371. doi: 10.3390/molecules26237371 (PMC8658876; doi:10.3390/molecules26237371)
Supplement: Supplementary file 1 [file molecules-26-07371-s001.zip › molecules-1460673-supplementary.pdf]

molecules

Supplementary Material

Optimized Liquid-Phase Exfoliation of Magnetic van der Waals Heterostructures: Towards the Single Layer and Deterministic Fabrication of Devices

Lucía Martín-Pérez [1] and Enrique Burzurí [1,2,*]

[1] IMDEA Nanociencia, Calle Faraday 9, Campus de Cantoblanco, 28049 Madrid, Spain; lucia.martin@imdea.org
[2] Departamento de Física de la Materia Condensada, Universidad Autónoma de Madrid, 28049 Madrid, Spain
* Correspondence: enrique.burzuri@uam.es

Index

1. Additional Scanning Electron Microscopy (SEM) images of bulk cylindrite. 2
2. Supplementary Table 1. Interpretation of the Raman spectra of cylindrite. 3
3. Additional AFM images and statistical details in Sample 1. 3
4. Additional AFM images and statistical details in Sample 3. 5
5. AFM characterization of Samples 2 and 4. 7
6. Additional AFM images and statistical details in Sample 5. 10
7. Statistical analysis of type 1 LPE cylindrite nanoflakes. 12
8. Additional SEM images of LPE cylindrite-based devices after DEP assembly. 13
9. Mathematical description of the equivalent R-CR electrical model. 14

1. Additional Scanning Electron Microscopy (SEM) images of bulk cylindrite.

Figure S1 shows the surface of the single cylindrite cylinder reported in Figure 1 (main manuscript). Figures S2, S3 and S4 show cylinder transverse-planes. A series of concentric layers can be clearly observed. The laminar nature of the material allows its exfoliation.

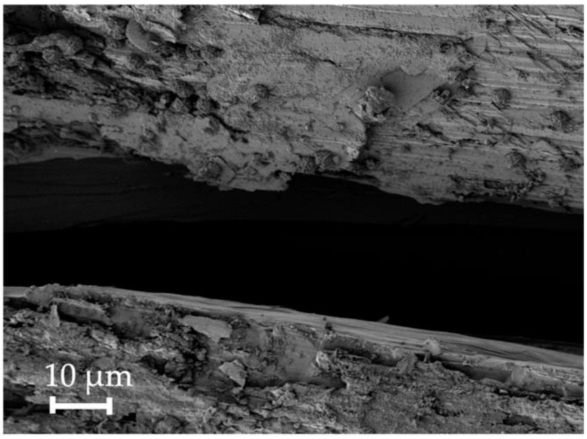

Figure S1. SEM image of a single cylindrite cylinder.

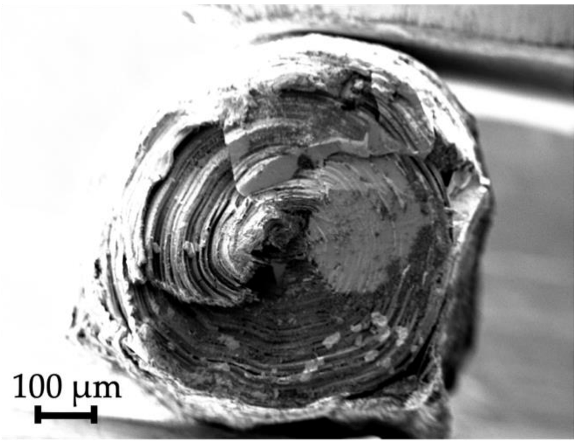

Figure S2. SEM image of the transversal-plane of a single cylindrite cylinder.

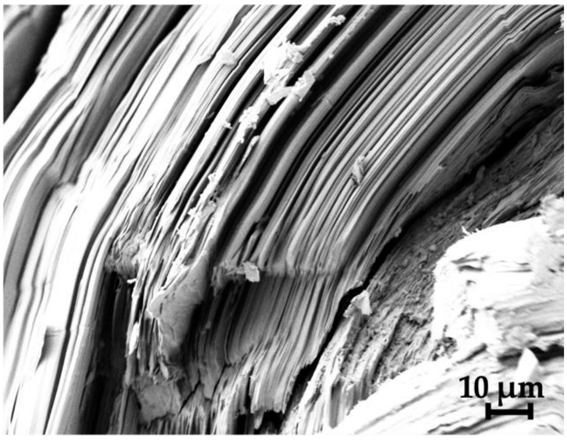

Figure S3. SEM image of the transversal-plane of a single cylindrite cylinder.

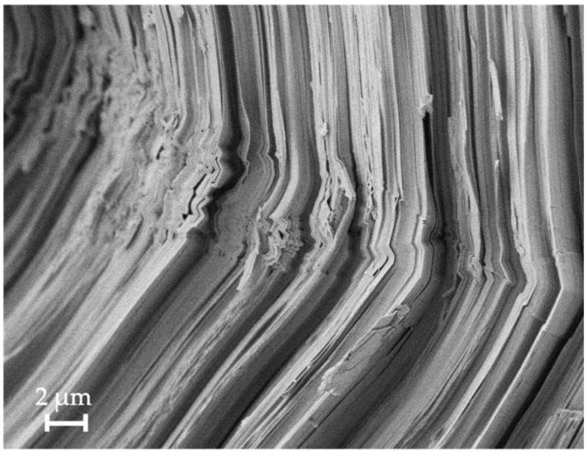

Figure S4. SEM image of the transversal-plane of a single cylindrite cylinder.

2. Supplementary Table 1. Interpretation of the Raman spectra of cylindrite.

Supplementary Table 1 shows the labelling and interpretation of the different Raman modes of cylindrite as reported for other mineral members of the sulfosalt family [38–41].

Table S1. Interpretation of the Raman spectra of cylindrite.

Raman Shift (cm^{-1})	Phonon Mode Attribution	Compound
70	Acoustic	PbS
90	A_{1g}	SnS
153	2nd order effect	SnS_2
	Transverse acoustic and transverse optical	PbS
189	Longitudinal optical	PbS
	E_g	SnS_2
242	Combination	PbS + SnS_2
305	A_{1g}	SnS_2

3. Additional AFM images and statistical details in Sample 1.

Figures S5 and S6 show the routine used to identify type 1 and type 2 flakes in Sample 1 shown in Figure 2a of the main manuscript. Type 1 flakes are blue-highlighted in Figure S5 whereas type 2 flakes are highlighted in Figure S6. The selection and statistical analysis is performed by Gwyddion and WSxM software [51].

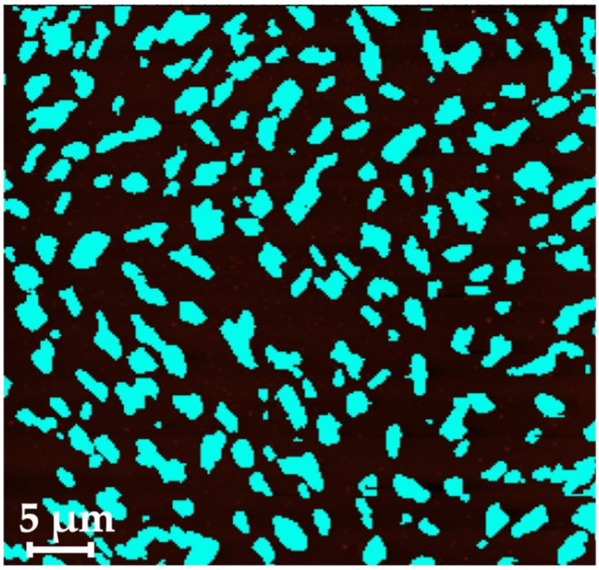

Figure S5. Type 1 flakes size selection of Sample 1 AFM image (50 × 50 µm^2), carried out with Gwyddion software.

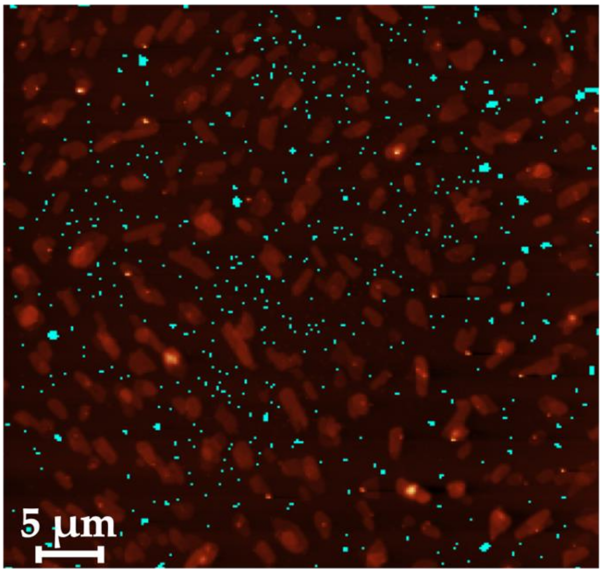

Figure S6. Type 2 flakes size selection of Sample 1 AFM image (50 × 50 µm^2), carried out with Gwyddion software.

Figures S7 and S8 show additional AFM images of cylindrite nanoflakes obtained in Sample 1 (Exfoliation parameters: 1 h sonication and 30 min centrifugation). The AFM samples are prepared by spin-coating the dispersion onto silicon wafer and dried in air.

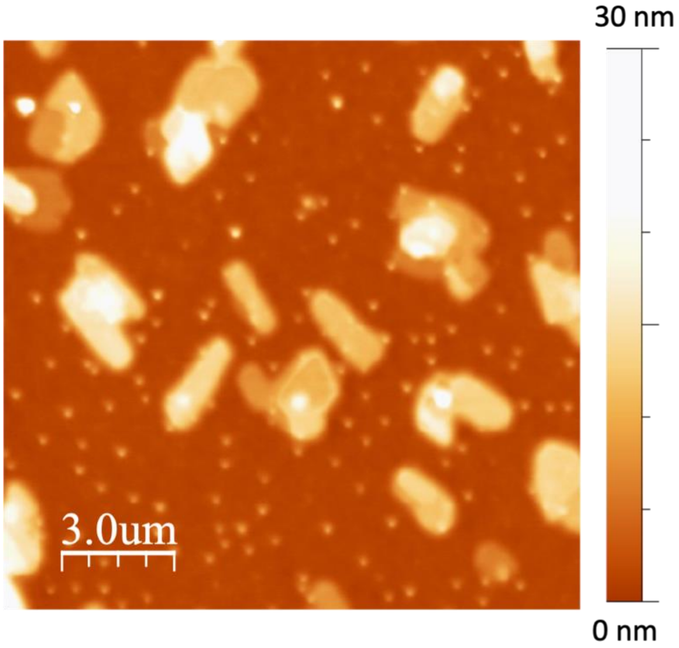

Figure S7. AFM topographic characterization of cylindrite nanoflakes obtained in Sample 1. Image dimension: 50 × 50 μm^2.

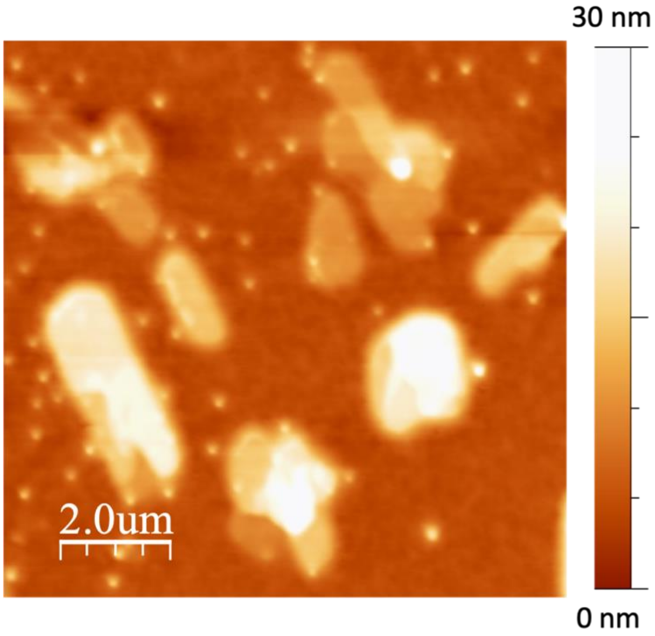

Figure S8. AFM topographic characterization of cylindrite nanoflakes obtained in Sample 1. Image dimension: 20 × 20 μm^2.

4. Additional AFM images and statistical details in Sample 3.

Figures S9 and S10 show the routine used to identify type 1 and type 2 flakes in Sample 3 shown in Figure 2f of the main manuscript. Type 1 flakes are green-highlighted in Figure S9 whereas type 2 flakes are highlighted in Figure S10. The selection and statistical analysis is performed by Gwyddion and WSxM software [51].

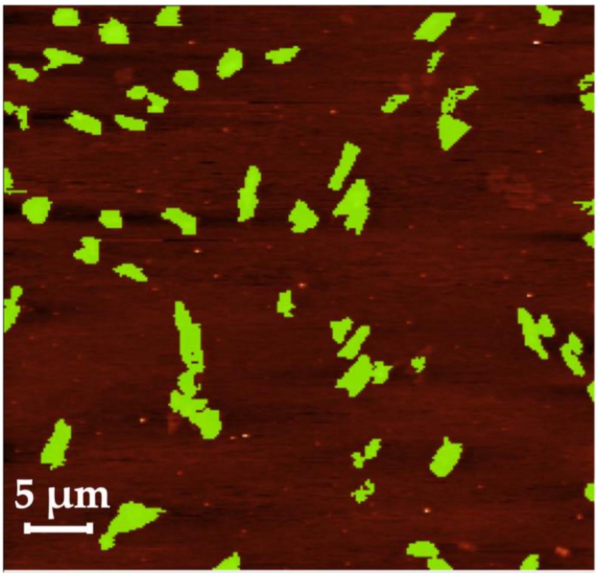

Figure S9. Type 1 flakes size selection of Sample 3 AFM image (50 × 50 µm^2), carried out with Gwyddion software.

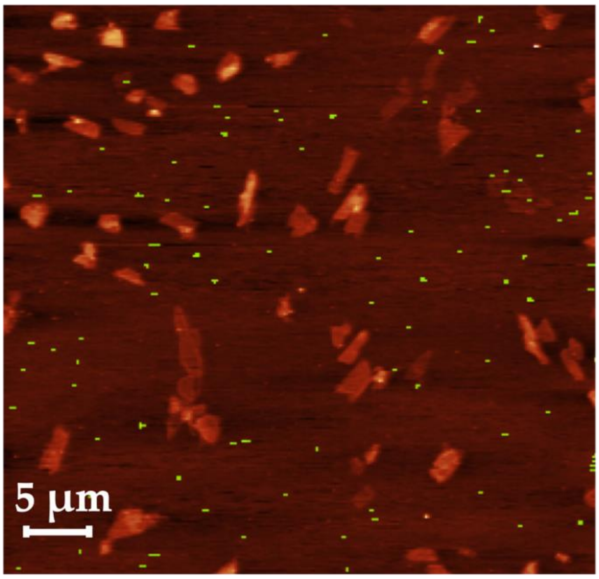

Figure S10. Type 2 flakes size selection of Sample 3 AFM image (50 × 50 µm^2), carried out with Gwyddion software.

Figures S11 and S12 shows additional AFM images of cylindrite nanoflakes obtained in Sample 3 (Exfoliation parameters: 2 h sonication and 1 h centrifugation). The AFM samples are prepared by spin-coating the dispersion onto silicon wafer and dried in air.

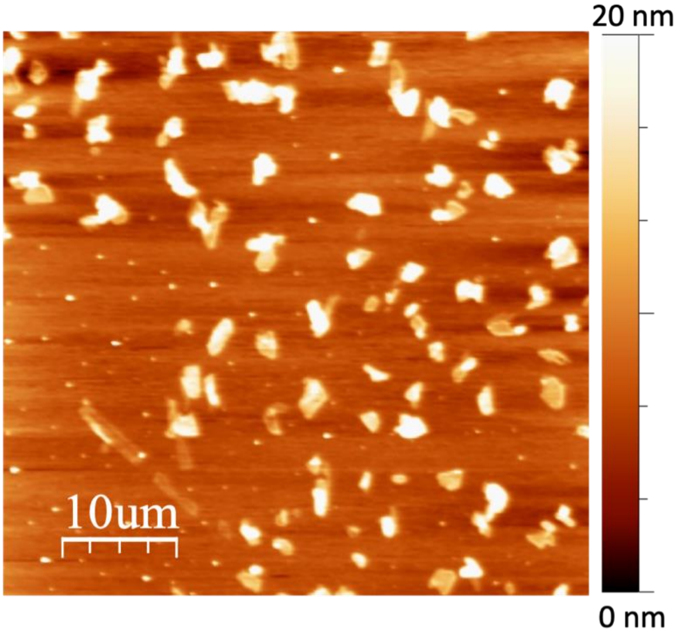

Figure S11. AFM topographic characterization of cylindrite nanoflakes obtained in Sample 3. Image dimension: 50 × 50 μm^2.

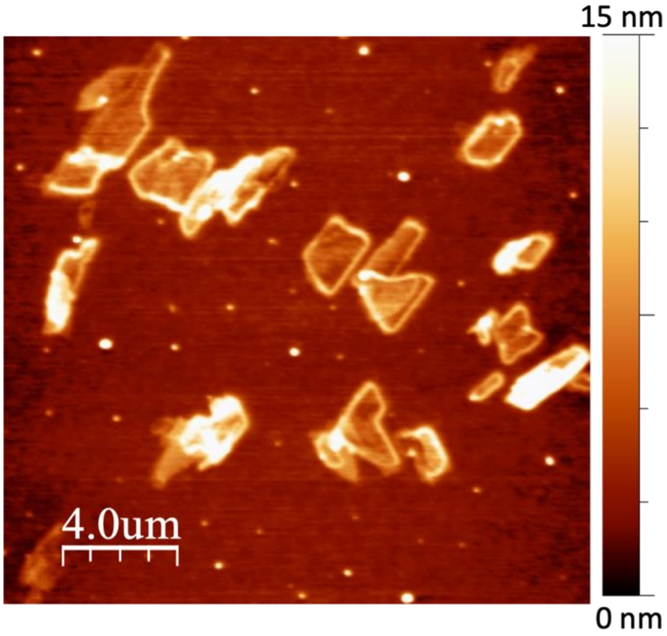

Figure S12. AFM topographic characterization of cylindrite nanoflakes obtained in Sample 3. Image dimension: 20 × 20 μm^2.

5. AFM characterization of Samples 2 and 4.

Figures S13, S14 and S15 show the AFM study of Sample 2. The nanoflakes present no morphology, size nor shape defined. The AFM samples are prepared by spin-coating the dispersion onto silicon wafer and dried in air.

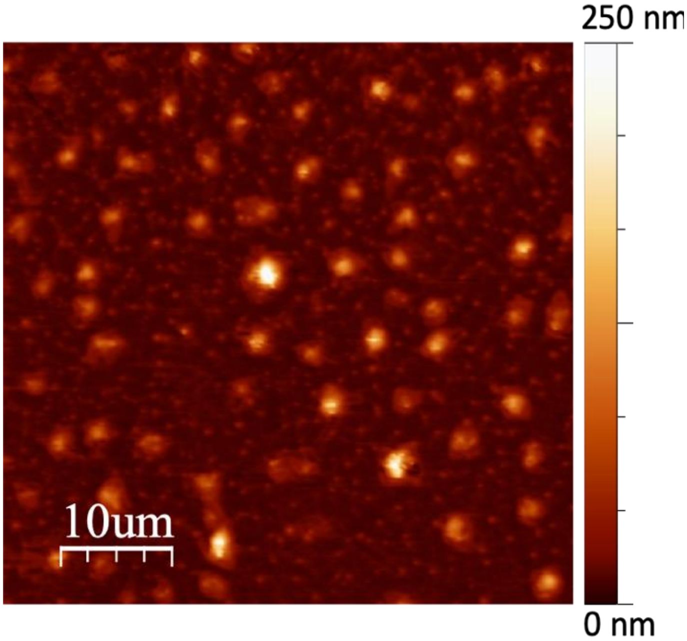

Figure S13. AFM topographic characterization of cylindrite nanoflakes obtained in Sample 2. Images dimension: 50 × 50 µm².

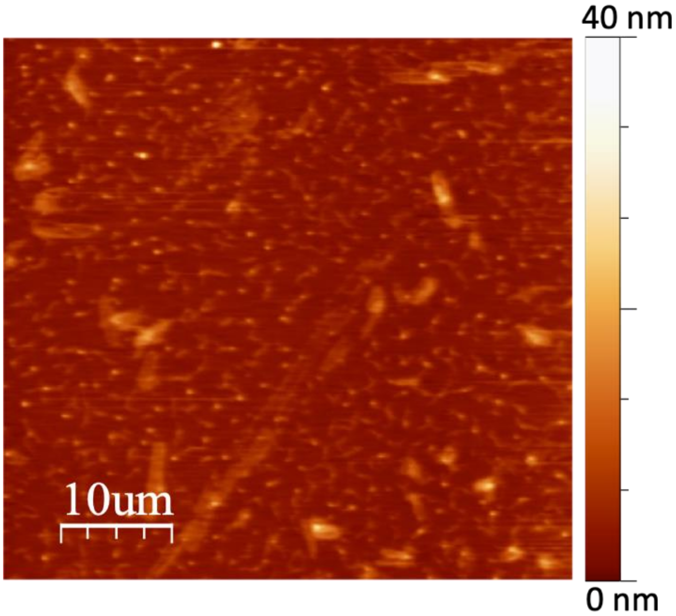

Figure S14. AFM topographic characterization of cylindrite nanoflakes obtained in Sample 2. Images dimension: 50 × 50 µm².

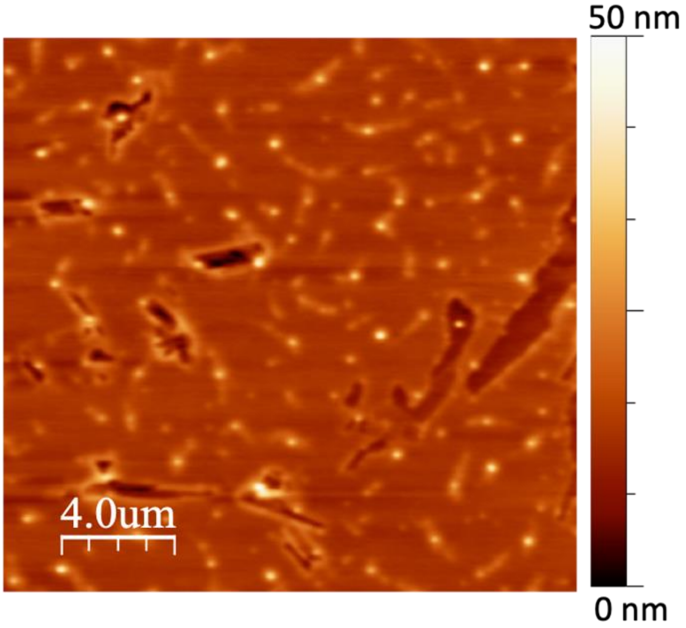

Figure S15. AFM topographic characterization of cylindrite nanoflakes obtained in Sample 2. Images dimension: 20 × 20 μm².

Figures S16, S17 and S18 show the AFM study of Sample 4. The nanoflakes present no morphology, size nor shape defined. The AFM samples are prepared by spin-coating the dispersion onto silicon wafer and dried in air.

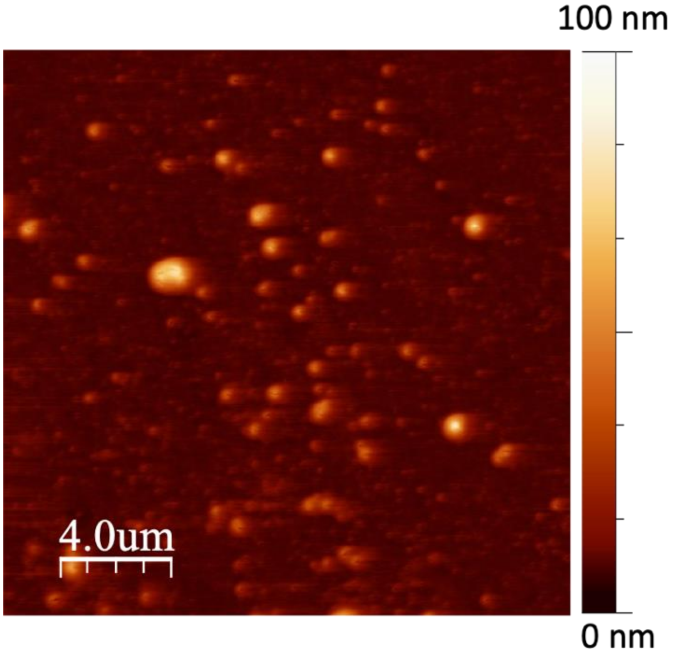

Figure S16. AFM topographic characterization of cylindrite nanoflakes obtained in Sample 2. Image dimension: 20 × 20 μm².

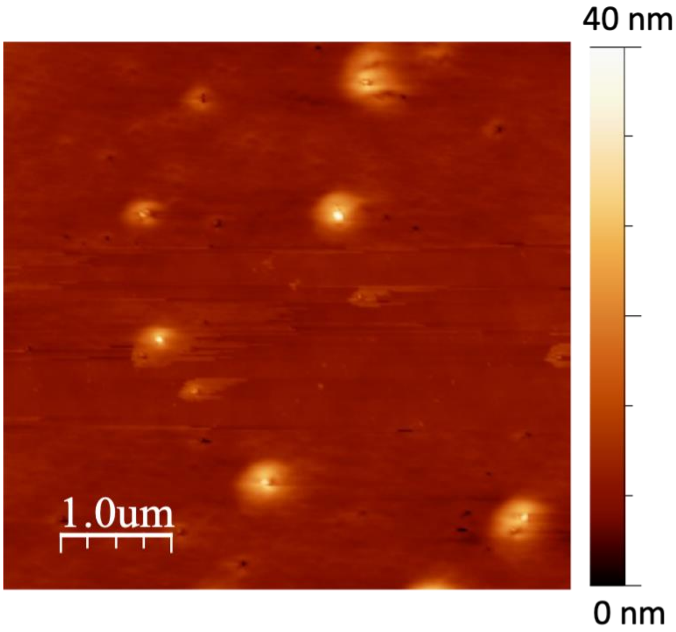

Figure S17. AFM topographic characterization of cylindrite nanoflakes obtained in Sample 4. Image dimension: 5 × 5 μm².

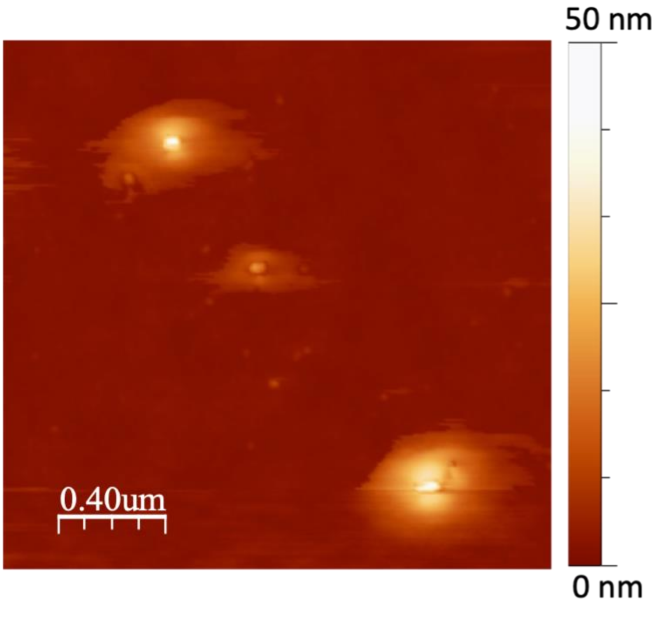

Figure S18. AFM topographic characterization of cylindrite nanoflakes obtained in Sample 4. Image dimension: 2 × 2 μm².

6. Additional AFM images and statistical details in Sample 5.

Figures S19 and S20 show the routine used to identify type 1 and type 2 flakes in Sample 5 shown in Figure 3a of the main manuscript. Type 1 flakes are green-highlighted in Figure S19 whereas type 2 flakes are highlighted in Figure S20. The selection and statistical analysis is performed by Gwyddion and WSxM software [51].

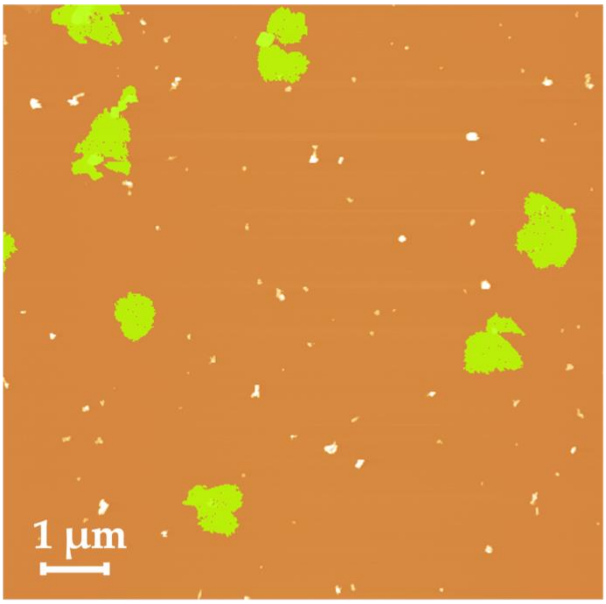

Figure S19. Type 1 flakes size selection of Sample 5 AFM image (10 × 10 µm^2), carried out with Gwyddion software.

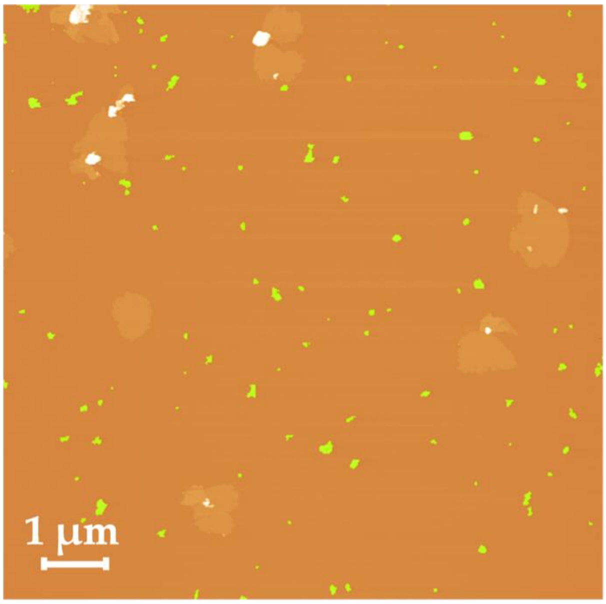

Figure S20. Type 2 flakes size selection of Sample 5 AFM image (50 × 50 µm^2), carried out with Gwyddion software.

Figures S21 and S22 show additional AFM images of cylindrite nanoflakes obtained in Sample 5 (Exfoliation parameters: 3 h sonication and 1 h centrifugation). The AFM samples are prepared by spin-coating the dispersion onto mica foil and dried in air.

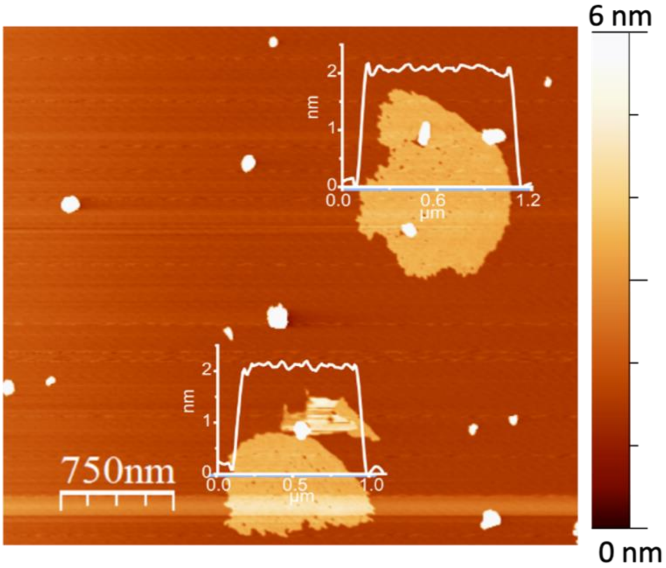

Figure S21. AFM topographic characterization of cylindrite nanoflakes obtained in Sample 5. Image dimension: 0.75 × 0.75 µm².

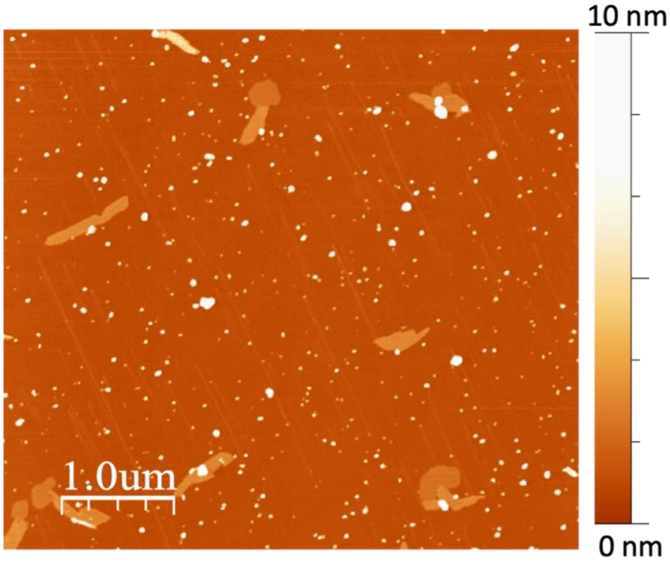

Figure S22. AFM topographic characterization of cylindrite nanoflakes obtained in Sample 5. Image dimension: 5 × 5 µm².

7. Statistical analysis of type 1 LPE cylindrite nanoflakes.

Figure S23 shows a direct comparison of the thickness in type 1 nanoflakes obtained in Samples 1, 3 and 5. The reduction of the flake thickness with increasing sonication time from sample 1 to sample 5 is clearly observed. The average flake thickness in sample 1 is 7.2 nm. In sample 3, 66% of the flakes have a thickness between 4.3 and 6.3 nm. Finally, the sample 5 measured flakes have thickness between 1 and 2 nm.

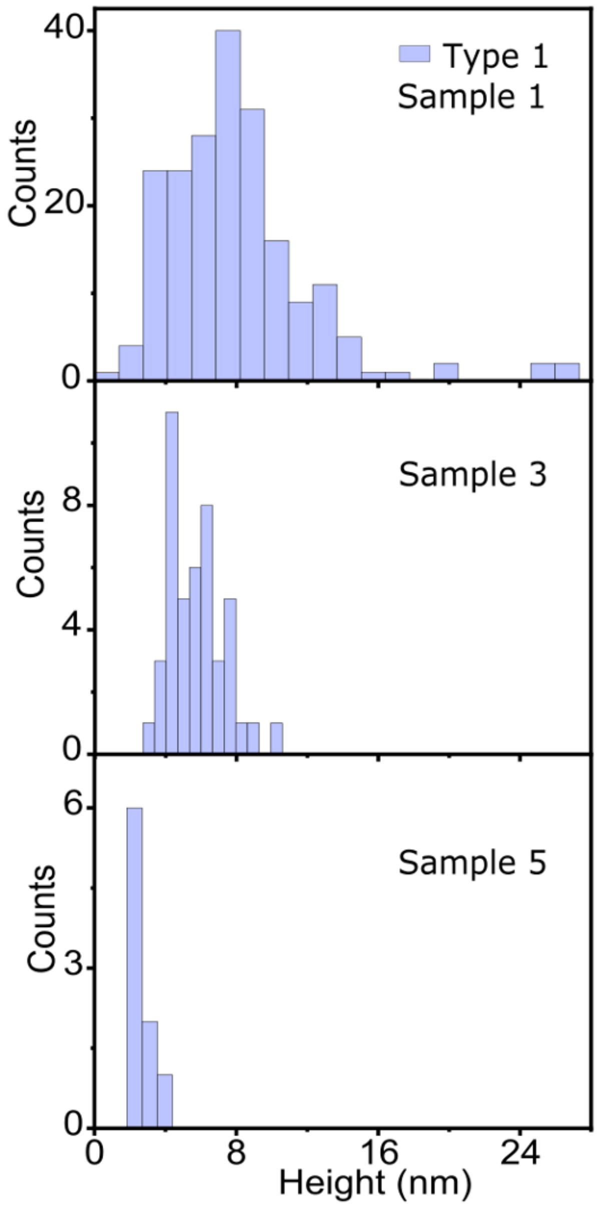

Figure S23. Statistical analysis of AFM height of type 1 LPE cylindrite nanoflakes obtained in Samples 1, 3 and 5.

8. Additional SEM images of LPE cylindrite-based devices after DEP assembly.

Additional SEM images of multi-electrode devices after DEP assembly are shown in Figures S24 and S25. Through this mechanism, the LPE cylindrite nanoflakes are transferred and positioned in the gap between the electrodes.

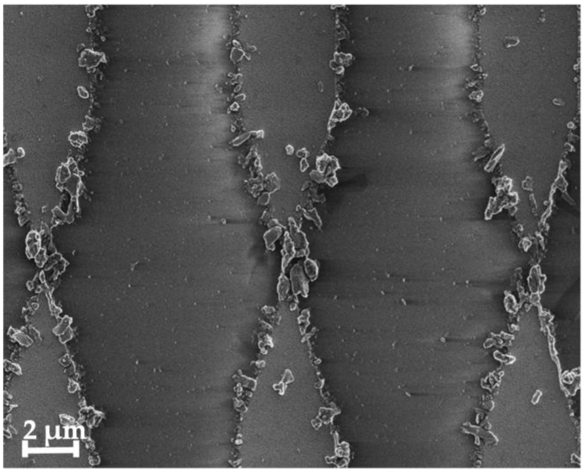

Figure S24. SEM images of multi-electrode device after DEP assembly.

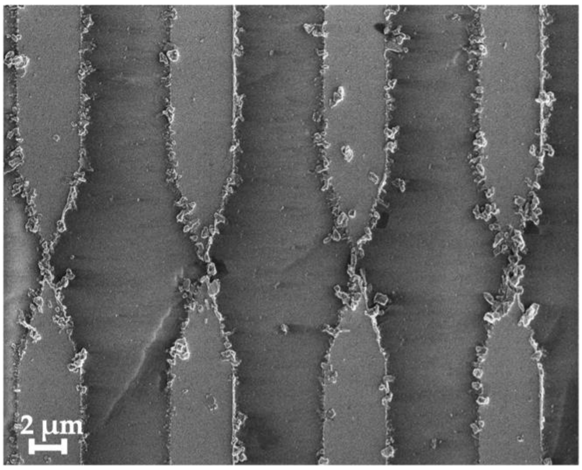

Figure S25. SEM images of multi-electrode device after DEP assembly.

9. Mathematical description of the equivalent R-CR electrical model.

The frequency response of the admittance Y^* observed in the main text can be approximately reproduced with a set of two parallel RC circuits connected in series. Each sub-circuit describes the contribution to charge transport of the grain boundaries (C_{gb}, R_{gb}) and the bulk crystal (C_c, R_c) [50]. The particular characteristics of the devices presented in the manuscript show that the experimental data can be fitted with a single parallel RC circuit (R_1, C_1) connected in series with a single R_2 resistance.

Here below, the mathematical description of the resulting circuit is explained in detail. The complex admittance Y^* (ω) of a circuit as a function of ω angular frequency can be written as the sum of conductance (G) and susceptance (B):

$$Y^*(\omega) = G(\omega) + i\,B(\omega) \tag{1}$$

The complex admittance of a resistive and a capacitive element are respectively:

$$Y^*(\omega) = 1/R \tag{2}$$

$$Y^*(\omega) = i\,\omega\,C \tag{3}$$

The complex admittance of the resulting parallel RC circuit is:

$$Y^* \, (\omega) = \frac{1}{R} + i \, \omega \, C \tag{4}$$

The addition of the two circuits in series would result in the total Y^* of the circuit:

$$Y^* \, (\omega) = \frac{1}{\frac{1}{\frac{1}{R_1} + i \, \omega \, C_1} + \frac{1}{\frac{1}{R_2}}} \tag{5}$$

From Equation (5) and (1) we obtain the *G* and *B* values of the circuit as:

$$G = \frac{1 + \ \omega^2 C_1^2 R_1 R_e}{(R_1 + R_2)(1 + \omega^2 R_e^2 C_1^2)} \tag{6}$$

$$B = \frac{\omega(C_1 R_1 R_e)}{R_2(R_1 + R_2)(1 + \omega^2 R_e^2 C_1^2)} \tag{7}$$

where R_e is the equivalent resistance of the circuit:

$$R_e = \frac{R_1 R_2}{R_1 + R_2} \tag{8}$$

The experimental *B* and *G* curves as a function of ω can be quantitatively reproduced by selecting an appropriate set of *R* and *C* parameters. The solid lines in Figures 4f and 4g in the main text are calculated by using Equations (6) and (7).
